# *ZmCCT* haplotype *H5* improves yield, stalk-rot resistance, and drought tolerance in maize

**DOI:** 10.3389/fpls.2022.984527

**Published:** 2022-08-15

**Authors:** Lixiu Tong, Mingzhu Yan, Mang Zhu, Jie Yang, Yipu Li, Mingliang Xu

**Affiliations:** ^1^State Key Laboratory of Plant Physiology and Biochemistry, College of Agronomy and Biotechnology, National Maize Improvement Center, Center for Crop Functional Genomics and Molecular Breeding, China Agricultural University, Beijing, China; ^2^Food Crops Research Institute, Xinjiang Academy of Agricultural Sciences, Urumqi, China; ^3^Agricultural College, Inner Mongolia Agricultural University, Hohhot, China

**Keywords:** *ZmCCT*, hybrids, yield, stalk-rot resistance, drought tolerance

## Abstract

The *ZmCCT* locus underlies both stalk-rot resistance and photoperiod sensitivity in maize (*Zea mays* L.). We previously introduced nine resistant *ZmCCT* haplotypes into seven elite but susceptible maize inbred lines (containing the haplotype *H1*) to generate 63 backcross families. Here, we continued backcrossing, followed by selfing, to develop 63 near-isogenic lines (NILs). We evaluated 22 of these NILs for stalk-rot resistance and flowering time under long-day conditions. Lines harboring the haplotype *H5* outperformed the others, steadily reducing disease severity, while showing less photoperiod sensitivity. To demonstrate the value of haplotype *H5* for maize production, we selected two pairs of NILs, 83B28*^H1^*/83B28*^H5^* and A5302*^H1^*/A5302*^H5^*, and generated F_1_ hybrids with the same genetic backgrounds but different *ZmCCT* alleles: 83B28*^H1^* × A5302*^H1^*, 83B28*^H1^* × A5302*^H5^*, 83B28*^H5^* × A5302*^H1^*, and 83B28*^H5^* × A5302*^H5^*. We performed field trials to investigate yield/yield-related traits, stalk-rot resistance, flowering time, and drought/salt tolerance in these four hybrids. 83B28*^H5^* × A5302*^H1^* performed the best, with significantly improved yield, stalk-rot resistance, and drought tolerance compared to the control (83B28*^H1^* × A5302*^H1^*). Therefore, the *ZmCCT* haplotype *H5* has great value for breeding maize varieties with high yield potential, stalk-rot resistance, and drought tolerance.

## Introduction

Increasing maize (*Zea mays* L.) output is crucial to meet the global demand for food security. However, both biotic and abiotic stress pose great threats to maize productivity. Breeding maize varieties with enhanced disease resistance and drought tolerance is the most effective and environmentally friendly way to ensure the security of maize production and supply.

Plants have evolved sophisticated defense responses against pathogens, including pattern-triggered immunity (PTI) and effector-triggered immunity (ETI). However, disease resistance is usually associated with a fitness penalty. In *Arabidopsis thaliana*, the *R* gene leads to a 5%–10% reduction in fitness in the absence of infection (Tian et al., 2003; Karasov et al., 2017). In crops, resistance (*R*) and susceptible (*S*) genes can play opposite roles in disease resistance and grain yield, with *S* genes decreasing disease resistance, but positively correlated with plant growth and development (Brown and Rant, 2013; Deng et al., 2017; Nelson et al., 2018). For example, *Mildew resistance locus O* (*mlo*) is derived from the loss of function of the dominant *S* gene (*MLO*), which confers durable, broad-spectrum resistance to powdery mildew in various species (Wang et al., 2014; Nekrasov et al., 2017). However, *mlo*-mediated resistance against powdery mildew is usually accompanied by a yield penalty (Büschges et al., 1997; Consonni et al., 2006), hampering the use of such resistance genes in crop breeding. Fortunately, the mildew-resistant mutant *Tamlo-R32* was recently created by genome editing and harbors an activated *Tonoplast monosaccharide transporter 3* (*TaTMT3B*) gene to rescue the *Tamlo*-related growth and yield penalty (Li et al., 2022). In addition, some ideal resistance genes appear to have no fitness cost and even increase yields (Li et al., 2017a; Wang et al., 2018). In rice, *IDEAL PLANT ARCHITECTURE 1* (*IPA1*) not only improves rice yields by reducing the formation of unproductive tillers to increase grains per panicle, but also enhances disease resistance by activating the pathogen defense gene *WRKY45* (*WRKY DNA-binding protein 45*; Wang et al., 2018). Thus, it is essential to evaluate the impact of a resistance gene on yield under normal and infected conditions before it can be appropriately applied for genetic improvement.

Pleiotropic genes are single genes (or a single locus) that affect two or more unrelated phenotypic traits (Solovieff et al., 2013) and numerous pleiotropic genes and QTLs have been reported in plants, whose functions range from growth and development to disease resistance (Liang et al., 2021). Most resistance genes in Arabidopsis have strong pleiotropic effects (Karasov et al., 2017). For example, the *Arabidopsis thaliana* typical coiled-coil (CC)-NBS-LRR (CNL) proteins *Resistance to Pseudomonas syringae 5* (*Rps5*) and *Resistance to P. syringae pv maculicola 1* (*Rpm1*) cause a fitness penalty when activated (Tian et al., 2003; Karasov et al., 2014). In maize, *ZmNF-Y3A* encodes a transcription factor that promotes early flowering and increases drought and heat stress tolerance by binding to different cis-elements in the promoters of various genes (Su et al., 2018).

In maize, a CCT (CONSTANS (CO), CO-LIKE, and TIMING OF CAB1) domain-containing transcription regulator gene, *ZmCCT*, was first reported to regulate flowering time under long-day conditions (Hung et al., 2012; Yang et al., 2013). Remarkably, *ZmCCT* was subsequently confirmed to be the causative gene at QTL-*qRfg1* associated with maize resistance to *Gibberella* stalk rot (Yang et al., 2010; Wang et al., 2017). The non-TE *ZmCCT* allele (lacking a transposable element [TE] in its promoter) is resistant to stalk rot and has photoperiod sensitivity under long-day conditions (Yang et al., 2013; Wang et al., 2017). In addition, the non-TE *ZmCCT* increases primary branch number, spike length (Xu et al., 2017), and crown root number (Zhang et al., 2018). A new *ZmCCT* allele was recently discovered that contains a 4.2-kilobase (kb) TE inserted in the intron of a non-TE *ZmCCT*; this allele shortens flowering time in low-latitude regions (Zhong et al., 2021). Phylogenetic analysis of genes encoding CCT proteins and their orthologs showed that the closest genes to *ZmCCT* are *Sb06g000570* in sorghum and *Ghd7* (*grain number, plant height, and heading date 7*) in rice (Xue et al., 2008; Yang et al., 2013). The CCT domain-containing gene *Ghd7* is involved in grain number, plant height, heading date (Xue et al., 2008), regulation of hormone metabolism, and biotic/abiotic stress responses (Weng et al., 2014). The finding that *ZmCCT* and *Ghd7* share highly similar genomic features prompted us to explore whether *ZmCCT* could also improve yield or other agronomic traits under various environmental conditions.

Marker-assisted selection (MAS) has been successfully used to breed disease-resistant lines (Zhao et al., 2012; Konlasuk et al., 2015; Xu et al., 2020). We previously introgressed nine non-TE *ZmCCT* haplotypes into seven elite inbred maize lines by MAS (Li et al., 2017b). In the current study, we selected 22 pairs of NILs derived from advanced backcross generations and evaluated their disease resistance and photoperiod sensitivity. We then focused on two pairs of NILs, 83B28*^H1^*/83B28*^H5^*, and A5302 *^H1^*/A5302*^H5^*, to prepare four F_1_ hybrids with the same genetic backgrounds but different *ZmCCT* haplotypes. These F_1_ hybrids were evaluated for their yield/yield-related traits, stalk-rot resistance, and flowering time under normal and biotic/abiotic stress conditions to assess the value of haplotype *H5* in the breeding of resistant maize varieties.

## Materials and methods

### Plant materials

We previously identified 15 haplotypes (*H1* to *H15*) at the *ZmCCT* locus. Of these, only haplotype *H1* contains a TE insertion in the promoter, whereas the other 14 lack a TE insertion (non-TE *ZmCCT*). Nine donor parents carrying different non-TE *ZmCCT* haplotypes (*H3*, *H4*, *H5*, *H6*, *H7*, *H12*, *H13*, *H14*, and *H15*) were previously crossed with and then backcrossed to seven elite inbred lines with haplotype *H1* (Zheng58, Chang7-2, 83B28, A5302, Jing24, F349, and Yu87-1) to generate 63 BC_5_F_1_ families (Li et al., 2017b). In the current study, we continued backcrossing twice to obtain 63 BC_7_F_1_ families, which were selfed and grown at the experimental station of China Agricultural University (Beijing, N39°54′, E116°24′). The BC_7_F_2_ families were planted in the Hainan winter nursery (Hainan, Sanya, N18°21′, E109°10′) in 2017/2018. After genotyping, BC_7_F_2_ plants from each family with homozygous haplotype *H1* or one of the non-TE *ZmCCT* haplotypes were continuously selfed to produce homozygous BC_7_F_3_ lines, which were grown at the experimental station of China Agricultural University to develop pairs of near-isogenic lines (NILs).

Due to limited resources, we chose five haplotypes (*H3*, *H5*, *H6*, *H12*, and *H13*) in the Zheng58 and Chang7-2 backgrounds and six haplotypes (*H3*, *H5*, *H6*, *H7*, *H12*, and *H13*) in the 83B28 and A5302 backgrounds for further study. We used the GoldenGate 6KSNP chip to survey the genome-wide SNPs. The genomic identities of two pairs of NILs, 83B28*^H1^*/83B28*^H5^*, and A5302*^H1^*/A5302*^H5^*, were estimated to be 96.86% and 93.71%, respectively. They were then crossed with one another to produce four F_1_ hybrids with the same genetic backgrounds but different *ZmCCT* haplotypes, including 83B28*^H1^* × A5302*^H1^*, 83B28*^H1^* × A5302*^H5^*, 83B28*^H5^* × A5302*^H1^*, and 83B28*^H5^* × A5302*^H5^*.

### Field experimental design

In 2018 and 2019, the NILs were grown in Beijing, China, and their stalk-rot resistance was investigated after artificial inoculation. Each pair of NILs was planted adjacent to each other, and different pairs of NILs were planted in a completely random design. Each NIL was planted in three rows, with 17 plants per row in three replicates. In 2020 and 2021, the 22 pairs of NILs were investigated for flowering time in Beijing, and due to the COVID-19 pandemic, each NIL was planted in two rows without replicates. For the four hybrids grown at different locations over the years, a randomized block design was implemented in the field trials with three replicates. Each hybrid was grown in three rows, with 17 plants per row.

### Preparation of *Fusarium graminearum* inoculum, field inoculation, and scoring of symptoms

The preparation of *Fusarium graminearum* inoculum and field inoculation was previously described in detail (Yang et al., 2010). The six-scale scoring system (0, 0.2, 0.4, 0.6, 0.8, and 1) was applied to investigate symptoms in the field. A disease severity index (DSI) was used to assess the stalk-rot severity for each line or hybrid. The DSI was calculated using the disease scales of all plants in the same line or hybrid as follows: DSI (%) = [∑(score × number of plants in score)]/total number of plants × 100.

### Investigation of flowering time and other agronomic traits

We investigated three flowering-related traits per plant, including days to heading (DTH), days to anthesis (DTA), and days to silking (DTS) for the NILs and hybrids in 2020 and 2021 in Beijing. All hybrids were investigated for yield and yield-related traits from 2018 to 2020. At the harvest stage, ears were collected from healthy plants and dried in the sun for ~2 weeks. Five ear-related traits were then measured: ear length (EL), bare tip length (BTL), ear diameter (ED), kernel row number (KRN), and kernel number per row (KNPR). After threshing, the kernels were stored at room temperature for open-air drying for at least 2 weeks, and the hundred kernel weight (HKW) and kernel weight per ear (KWPE) were measured. Two plant morphological traits were also investigated from 2019 to 2021: plant height (PH) and ear height (EH).

### Investigation of plant tolerance to drought and salt stress

The four hybrids were also planted in Xinjiang (from 2018 to 2020) and Ningxia (2018 and 2019), China to investigate yield and yield-related traits under drought and salt stress conditions, respectively. Xinjiang is an ideal place to test the drought tolerance of maize. Moreover, we employed drip irrigation under a mulch film, making it easy to control irrigation. For well-watered (control) conditions, the plants were irrigated regularly as needed. For drought stress conditions, the amount of irrigation water was reduced by half during the entire growth period compared to well-watered conditions, except for the first irrigation at the time of sowing. The Northeast of Ningxia Plain contains mostly alkaline soil, making it an excellent place to test plant tolerance to salt stress. We tested the soil salinity before sowing in 2018 and 2019. The salinity of 0–25-cm soil was 1.10% and 1.11% in 2018 and 2019, respectively, with a soil pH of 8.6 in 2019, indicating that the chosen site was an alkaline field with salt stress conditions.

### Statistical analysis

For each pair of NILs, the two NILs differed solely at the *ZmCCT* locus, i.e., the resistant allele without the TE versus the susceptible allele with the TE insertion. Significant differences in DSI and flowering time between two NILs were analyzed by *t*-test. For the hybrids, all data were analyzed by one-way analysis of variance (ANOVA) with Duncan’s test and Tamhane’s test using IBM SPSS Statistics (version 21.0.0.0).

## Results

### Evaluation of stalk-rot resistance and flowering time in the NILs

**Figure 1:**
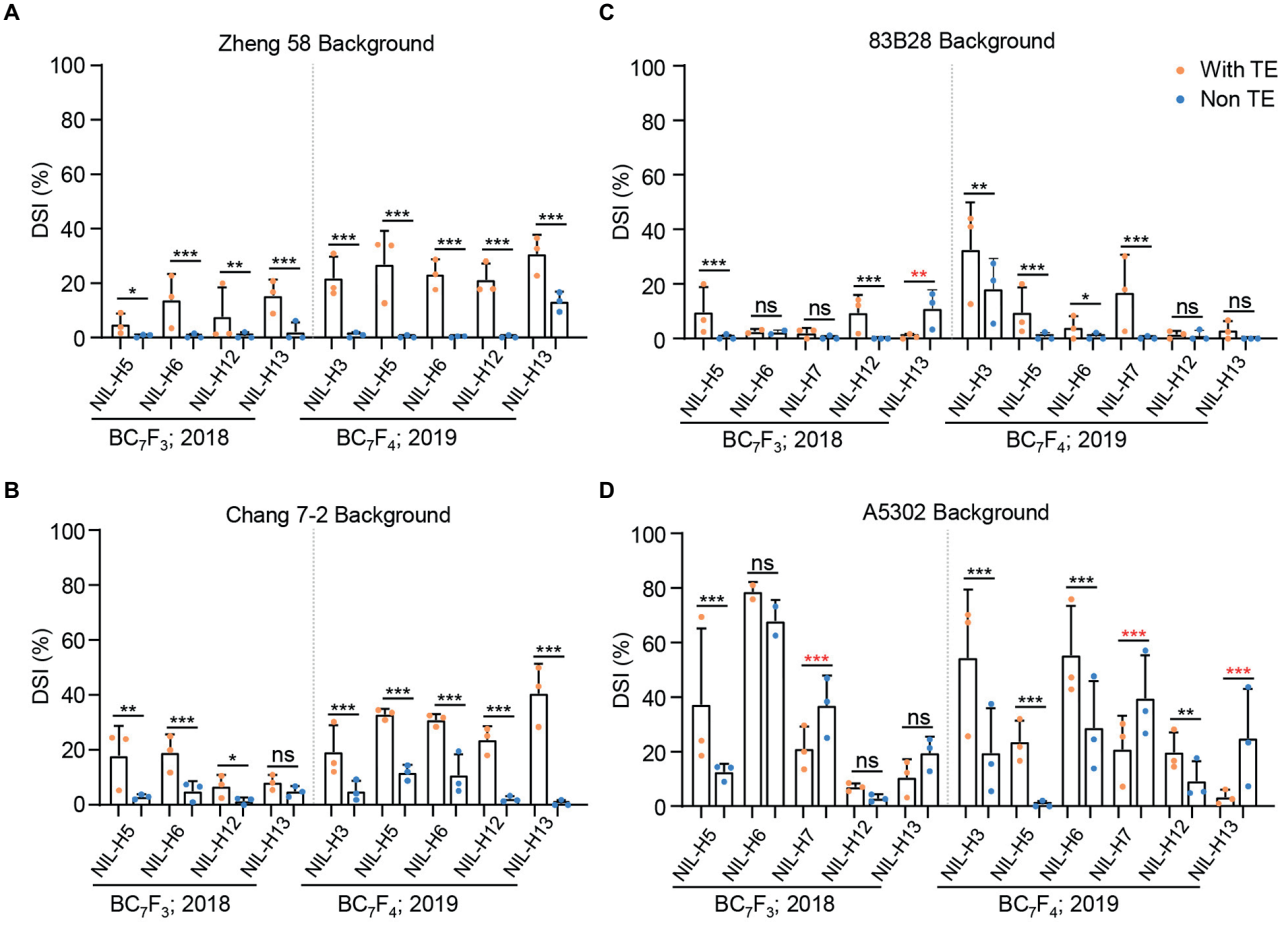
DSI of 22 NILs in four different backgrounds in 2018–2019 (Beijing). Five pairs of NILs include with TE *ZmCCT* homozygotes, and non-TE *ZmCCT* homozygotes in Zheng 58 **(A)**, Chang 7–2 **(B)**, 83B28 **(C)**, and A5302 **(D)** background were investigated for DSI. NILs include with TE *ZmCCT* homozygotes: *H1/H1*, and non-TE *ZmCCT* homozygotes: *H3/H3*, *H5/H5*, *H6/H6*, *H7/H7*, *H12/H12,* or *H13/H13* in different background were investigated for DSI. Values are mean ± standard deviation (s.d.). Asterisks indicate significant differences between *H1/H1* plants and the other genotypes (two-tailed paired Student’s *t-test*, ^*^p < 0.05, ^**^p < 0.01, ^***^p < 0.001, ns, not significant).

We previously determined that all BC_3_F_1_ and BC_5_F_1_ plants with the non-TE *ZmCCT* showed dramatically reduced DSI (Li et al., 2017b). Of the NILs developed in the current study, we selected 22 lines to further examine stalk-rot resistance and flowering time in 2018 and 2019. Plants in the Zheng58 background with the non-TE *ZmCCT* haplotypes (except H13) showed DSI values of almost zero in both 2018 and 2019, i.e., almost no plants were diseased. By contrast, the haplotype *H13* decreased the DSI by 13.32% and 17.45% in 2018 and 2019, respectively (Figure 1A). In the Chang7-2 background, the non-TE *ZmCCT* haplotypes also dramatically decreased the DSI values, with average decreases of 9.43% and 23.38% in 2018 and 2019, respectively (Figure 1B). However, in the 83B28 and A5302 backgrounds, five non-TE *ZmCCT* haplotypes (*H5*, *H6*, *H7*, *H12*, and *H13*) tested in 2018 and six (*H3*, *H5*, *H6*, *H7*, *H12*, and *H13*) tested in 2019 showed a certain degree of variation in stalk-rot resistance (Figures 1C,D), with only haplotypes *H3* and *H5* steadily reducing the DSI. In 2019, haplotype *H3* reduced the DSI by 14.42% in the 83B28 background and 34.81% in the A5302 background. Likewise, in 2018 and 2019, haplotype *H5* reduced the DSI by 9.20% and 8.77% in the 83B28 background and 24.74% and 22.87% in the A5302 background, respectively (Figures 1C,D). These results indicate that the reduction in the severity of maize stalk-rot disease is determined by a combination of the genetic background and *ZmCCT* haplotype.

**Figure 2:**
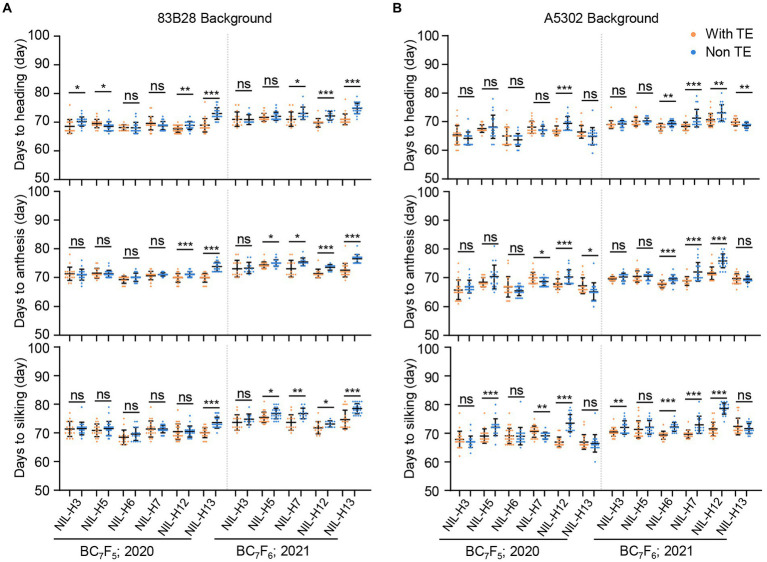
Twenty-two pairs of NILs in 83B28 **(A)** and A5302 **(B)** background were investigated for flowering time in 2020–2021 Beijing under long-day conditions. The genotypes of every pair of NILs were with TE *ZmCCT* homozygotes: *H1/H1*, and non-TE *ZmCCT* homozygotes: *H3/H3*, *H5/H5*, *H6/H6*, *H7/H7*, *H12/H12*, or *H13/H13*. Values are mean ± standard deviation (s.d.). Asterisks indicate significant differences between *H1/H1* plants and the other genotypes (two-tailed paired Student’s *t-test*, ^*^p < 0.05, ^**^p < 0.01, ^***^p < 0.001, ns, not significant).

We also investigated the flowering-related traits DTH, DTA, and DTS in the same sets of NILs. In the Zheng58 background, all non-TE *ZmCCT* haplotypes were more sensitive to long-day conditions than the TE haplotype *H1* and delayed DTH, DTA, and DTS in Beijing by an average of 5.0, 7.0, and 10.6 days, respectively (Supplementary Figure 1A). Notably, the non-TE *ZmCCT* haplotypes in the Chang7-2 background were even more sensitive to long-day conditions than those in the Zheng58 background, resulting in further delays in DTH, DTA, and DTS by an average of 10.6, 11.6, and 13.3 days, respectively (Supplementary Figure 1B). By contrast, non-TE *ZmCCT* haplotypes in the 83B28 and A5302 backgrounds were less sensitive to long-day conditions in Beijing and did not show significant differences in most flowering-related traits. However, there were some subtle differences among the non-TE *ZmCCT* haplotypes, with haplotypes *H3* and *H5* showing no or less sensitivity to photoperiod in the 83B28 and A5302 backgrounds during 2020 and 2021 (Figures 2A,B).

Of the non-TE *ZmCCT* haplotypes tested, *H5* performed the best, with high and stable stalk-rot resistance as well as less or no photoperiod sensitivity in the 83B28 and A5302 backgrounds, suggesting it would be valuable for breeding stalk-rot-resistant varieties.

### Stalk-rot resistance and flowering times of four F_1_ hybrids

**Figure 3:**
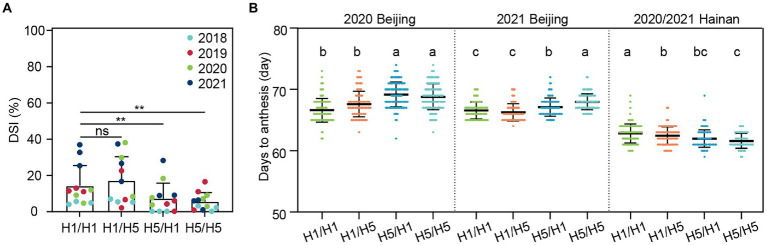
Evaluation of DSI and flowering time of four hybrids. In 2018–2021, the DSI **(A)** was investigated after artificial inoculated, each spot represents one replication of field trial (two-tailed paired Student’s t-test, **p < 0.01, ns, not signifcant). The flowering time **(B)** of four hybrids was investigated in 2020–2021 Beijing and 2020/2021 Hainan, which represents long-day conditions and short-day conditions, respectively. H1/H1, H1/H5, H5/H1, and H5/H5 are genotypes of four hybrids representing 83B28*^H1^* × A5302*^H1^*, 83B28*^H1^* × A5302*^H5^*, 83B28*^H5^* × A5302*^H1^*, and 83B28*^H5^* × A5302*^H5^*, respectively. Values are mean ± standard deviation (s.d.). Different letters indicate significant differences among hybrids (*p* < 0.05, one-way ANOVA, Tamhane test).

We used two pairs of NILs, 83B28*^H1^*/83B28*^H5^* and A5302*^H1^*/A5302*^H5^*, to generate four F_1_ hybrids. Overall, the F_1_ hybrids exhibited higher stalk-rot resistance than the parental lines in both 2018 and 2019, presumably due to heterosis. Among the four F_1_ hybrids, 83B28*^H5^* × A5302*^H1^* and 83B28*^H5^* × A5302*^H5^* only produced a few diseased plants. Compared to 83B28*^H1^* × A5302*^H1^*, these two hybrids showed significantly lower DSI values, with an average reduction of 6.98% and 8.68%, respectively (Figure 3A; Table 1). However, there was no significant difference in the DSI values between 83B28*^H1^* × A5302*^H5^* and 83B28*^H1^* × A5302*^H1^* (Figure 3A). These findings suggest that the maternal parental line plays a decisive role in stalk-rot resistance and that the homozygous *ZmCCT* haplotype *H5/H5* does not further increase stalk-rot resistance compared to the heterozygous *ZmCCT* haplotype *H5/H1*.

**Table 1:** Two-way ANOVA of DSI among different genotypes and years.

Source	SS	*df*	MS	F	*p*-value	F crit
Genotype	1107.882	3	369.29	7.58	5.73E-04	2.90
Year	1700.355	3	566.78	11.64	2.56E-05	2.90
Interact	1097.664	9	121.96	2.50	2.69E-02	2.19
Interclass	1558.021	32	48.69			
Total	5463.922	47				

We also investigated the flowering-related traits of the four hybrids under long-day (Beijing, 2020) and short-day (Hainan, 2020/2021) conditions. In Beijing, the *H5*-containing parental lines 83B28*^H5^* and A5302*^H5^* did not show detectible photoperiod sensitivity (Figures 2A,B), whereas the hybrids 83B28*^H5^* × A5302*^H1^* and 83B28*^H5^* × A5302*^H5^* showed 1.4- and 1.8-day delays in flowering time (average of DTH, DTA, and DTS), respectively, compared to 83B28*^H1^* × A5302*^H1^*. By contrast, the three flowering-related traits of the 83B28*^H1^* × A5302*^H5^* hybrid did not significantly differ from those of 83B28*^H1^* × A5302*^H1^* (Figure 3B; Supplementary Figure 2). In Hainan, the hybrid with two *H5* alleles (83B28*^H5^* × A5302*^H5^*) showed earlier flowering times than the hybrids with a single *H5* (83B28*^H5^* × A5302*^H1^* and 83B28*^H1^* × A5302*^H5^*), while 83B28*^H1^* × A5302*^H1^* showed the latest flowering time (Figure 3B; Supplementary Figure 2). Taken together, these results indicate that hybrids with the heterozygous genotype at *ZmCCT* (*H5/H1*) can help meet the breeding goal of improved maize stalk-rot resistance with little or no photoperiod sensitivity under long-day conditions.

### The genetic effects of *H5* on yield of the hybrids under infected conditions

**Figure 4:**
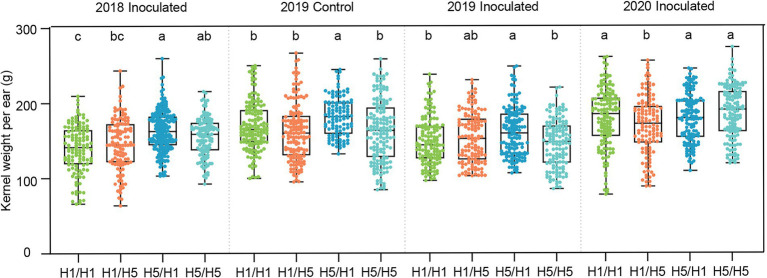
The kernel weight per ear of hybrids in Beijing under control and biotic stress. H1/H1, H1/H5, H5/H1, and H5/H5 are genotypes of four hybrids represent 83B28*^H1^* × A5302*^H1^*, 83B28*^H1^* × A5302*^H5^*, 83B28*^H5^* × A5302*^H1^*, and 83B28*^H5^* × A5302*^H5^*, respectively. Boxes show mean and quartile, whiskers represent max to min. Different letters indicate significant differences among hybrids (*p* < 0.05, one-way ANOVA, Tamhane’s test and Duncan’s test).

To test the genetic effect of the haplotype *H5* on yield under infected conditions, we planted the four hybrids in Beijing and performed artificial inoculation in the field, with no inoculation as a control. In 2018, hybrids 83B28*^H5^* × A5302*^H1^* and 83B28*^H5^* × A5302*^H5^* had significantly higher kernel weight per ear (KWPE) than 83B28*^H1^* × A5302*^H1^* under infected conditions, with increases of 16.6% and 11.4%, respectively, whereas the KWPE of 83B28*^H1^* × A5302*^H5^* did not significantly differ from that of 83B28*^H1^* × A5302*^H1^* (Figure 4). To confirm these findings, we repeated the experiment in Beijing in 2019 and added a no inoculation control. Compared to 83B28*^H1^* × A5302*^H1^*, 83B28*^H5^* × A5302*^H1^* showed a significantly higher KWPE under both infected and non-infected conditions, increasing by 7.4% and 8.0%, respectively. By contrast, the other two hybrids, 83B28*^H1^* × A5302*^H5^* and 83B28*^H5^* × A5302*^H5^*, did not show significant improvements in KWPE. In 2020, the KWPE of 83B28*^H5^* × A5302*^H1^* was not significantly improved compared with 83B28*^H1^* × A5302*^H1^*, likely due to environmental effects (Figure 4).

Consistent with its yield, the 83B28*^H5^* × A5302*^H1^* hybrid generally performed better than the control (83B28*^H1^* × A5302*^H1^*) for yield-related traits, including ear length (EL), bare tip length (BTL), ear diameter (ED), kernel row number (KRN), kernel number per row (KNPR), and hundred kernel weight (HKW). In 2018, 83B28*^H5^* × A5302*^H1^* had greater EL, KNPR, and ED values than those of 83B28*^H1^* × A5302*^H1^* (Supplementary Figure 3). The hybrid 83B28*^H5^* × A5302*^H5^* had better BTL, ED, and KRN but worse HKW values than the control (Supplementary Figure 3). In 2019, 83B28*^H5^* × A5302*^H1^* had greater ED and HKW values than 83B28*^H1^* × A5302*^H1^* under normal conditions but showed no significant differences in any yield-related traits compared to 83B28*^H1^* × A5302*^H1^* under infected conditions (Supplementary Figure 3). By contrast, 83B28*^H1^* × A5302*^H5^* and 83B28*^H5^* × A5302*^H5^* showed better BTL and KRN but worse EL, KPR, and HKW values than the control under both infected and normal conditions (Supplementary Figure 3). In 2020, 83B28*^H5^* × A5302*^H1^* had higher EL and KPR values but lower ED, KRN, and HKW values than those of 83B28*^H1^* × A5302*^H1^*, resulting in no difference in KWPE (Supplementary Figure 3; Figure 4). Taken together, these findings indicate that 83B28*^H5^* × A5302*^H1^* performed better than the other hybrids in terms of yield and yield-related traits.

### The genetic effects of *H5* on yield under abiotic stress conditions

**Figure 5:**
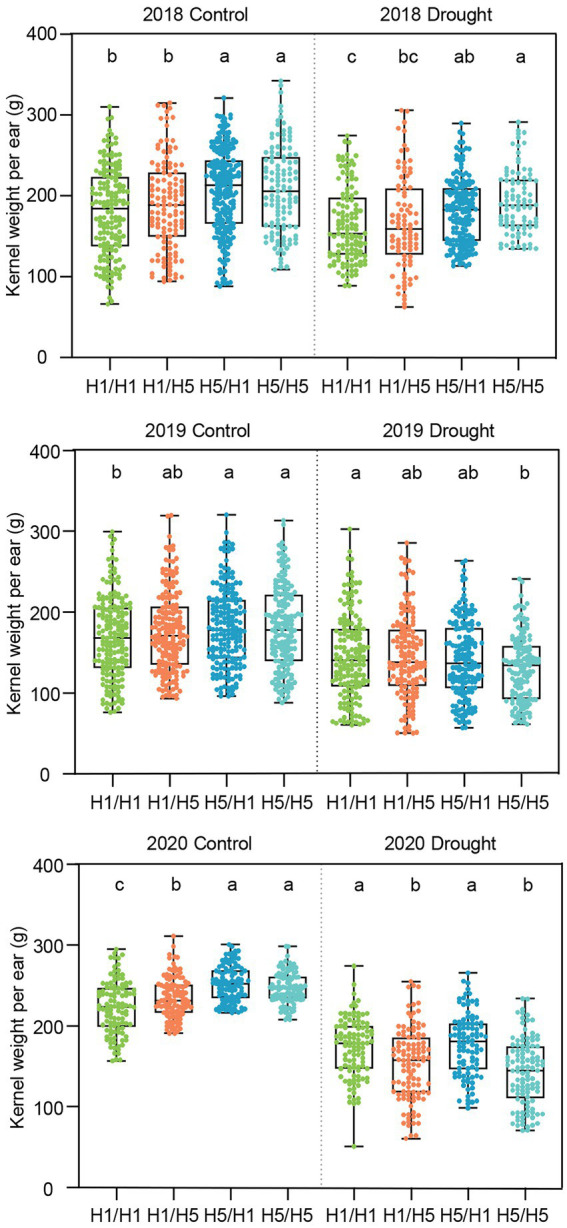
The kernel weight per ear of hybrids in 2018–2020 Xinjiang under control and drought stress. Figures above show the KWPE of hybrids in 2018, 2019, and 2020, respectively. H1/H1, H1/H5, H5/H1, and H5/H5 are genotypes of four hybrids represent 83B28*^H1^* × A5302*^H1^*, 83B28*^H1^* × A5302*^H5^*, 83B28*^H5^* × A5302*^H1^*, and 83B28*^H5^* × A5302*^H5^*, respectively. Boxes show mean and quartile, whiskers represent max to min. Different letters indicate significant differences among hybrids (*p* < 0.05, one-way ANOVA, Duncan’s test, and Tamhane’s test).

We tested the yield and yield-related traits of the hybrids under drought stress conditions in Xinjiang from 2018 to 2020 and salt stress conditions in Ningxia in 2018 and 2019. Under normal irrigation conditions in Xinjiang from 2018 to 2020, 83B28*^H5^* × A5302*^H1^* and 83B28*^H5^* × A5302*^H5^* showed significantly higher KWPE compared to 83B28*^H1^* × A5302*^H1^* and 83B28*^H1^* × A5302*^H5^* (Figure 5). Under drought stress, 83B28*^H5^* × A5302*^H1^* showed the best KWPE among the four hybrids in 2018, with an increase of 9.7% compared to the control (83B28*^H1^* × A5302*^H1^*), but with no significant difference in this value in 2019 and 2020. The hybrid 83B28*^H5^* × A5302*^H5^* showed a higher KWPE value in 2018 but lower values in 2019 and 2020 compared to the control. The KWPE of 83B28*^H1^* × A5302*^H5^* did not significantly differ from the control value from 2018 to 2019 but was lower than the control value in 2020 (Figure 5). Overall, 83B28*^H5^* × A5302*^H1^* performed the best among the four hybrids.

**Figure 6:**
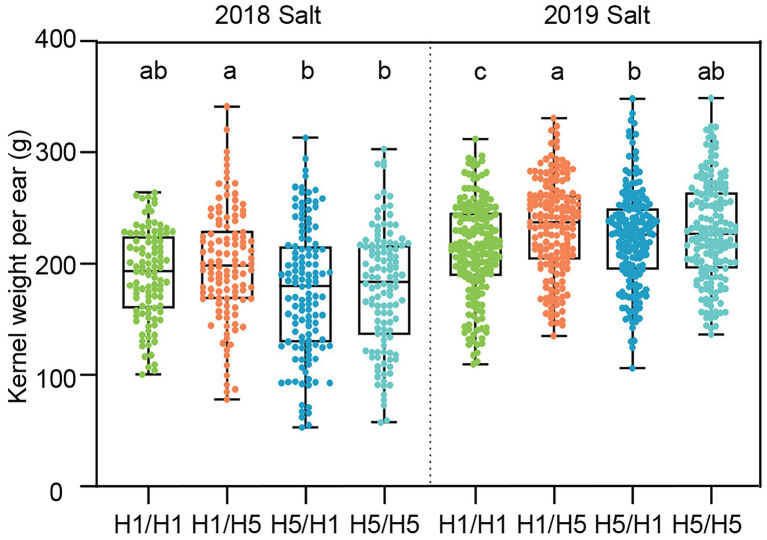
The yield-related traits of hybrids in 2018–2019 Ningxia under salt stress conditions. H1/H1, H1/H5, H5/H1, and H5/H5 are genotypes of four hybrids represent 83B28*^H1^* × A5302*^H1^*, 83B28*^H1^* × A5302*^H5^*, 83B28*^H5^* × A5302*^H1^*, and 83B28*^H5^* × A5302*^H5^*, respectively. Boxes show mean and quartile, whiskers represent max to min. Different letters indicate significant differences among hybrids (*p* < 0.05, one-way ANOVA, Duncan’s test, and Tamhane’s test).

Under salt stress in Ningxia, 83B28*^H5^* × A5302*^H1^* and 83B28*^H5^* × A5302*^H5^* had lower KWPE values in 2018 but higher values in 2019 compared to 83B28*^H1^* × A5302*^H1^*. The KWPE of 83B28*^H1^* × A5302*^H5^* did not significantly differ from the control in 2018 but was higher than the control value in 2019 (Figure 6). Unlike under *F. graminearum* inoculation and drought stress conditions, 83B28*^H1^* × A5302*^H5^* showed the best performance under salt stress conditions, suggesting that the haplotype *H5* employs different mechanisms in response to different stress conditions.

When comparing yield-related traits among the four hybrids in Xinjiang, each hybrid showed similar trends under normal and drought conditions (Supplementary Figure 4). In 2018, 83B28*^H5^* × A5302*^H1^* had significantly higher EL, lower BTL, and higher KPR than 83B28*^H1^* × A5302*^H1^* under both normal and drought conditions (Supplementary Figure 4). Similarly, 83B28*^H5^* × A5302*^H5^* showed better EL, BTL, KPR, and ED values, whereas 83B28*^H1^* × A5302*^H5^* only showed improved ED and KRN values (Supplementary Figure 4). In 2019, 83B28*^H5^* × A5302*^H1^* showed higher HKW than 83B28*^H1^* × A5302*^H1^* under drought stress but no significant improvements in any other yield-related trait (Supplementary Figure 4). In 2020, 83B28*^H5^* × A5302*^H1^* had higher EL, lower BTL, and higher HKW but lower ED compared to 83B28*^H1^* × A5302*^H1^* under drought stress, while 83B28*^H5^* × A5302*^H5^* showed higher EL and lower BTL but lower ED vs. the control (Supplementary Figure 4). Finally, 83B28*^H1^* × A5302*^H5^* had a similar performance for yield-related traits compared to the control (83B28*^H1^* × A5302*^H1^*; Supplementary Figure 4).

We also compared the yield-related traits of the hybrids under salt stress in Ningxia. The hybrid 83B28*^H5^* × A5302*^H1^* had significantly smaller ED than 83B28*^H1^* × A5302*^H1^*, and 83B28*^H1^* × A5302*^H5^* had significantly higher KRN than 83B28*^H1^* × A5302*^H1^* (Supplementary Figure 5). For the remaining yield-related traits, no significant differences were detected among the four hybrids in 2018 and 2019 (Supplementary Figure 5). These yield-related traits underlie the KWPE performance of the four hybrids.

### Morphological traits of the hybrids

**Figure 7:**
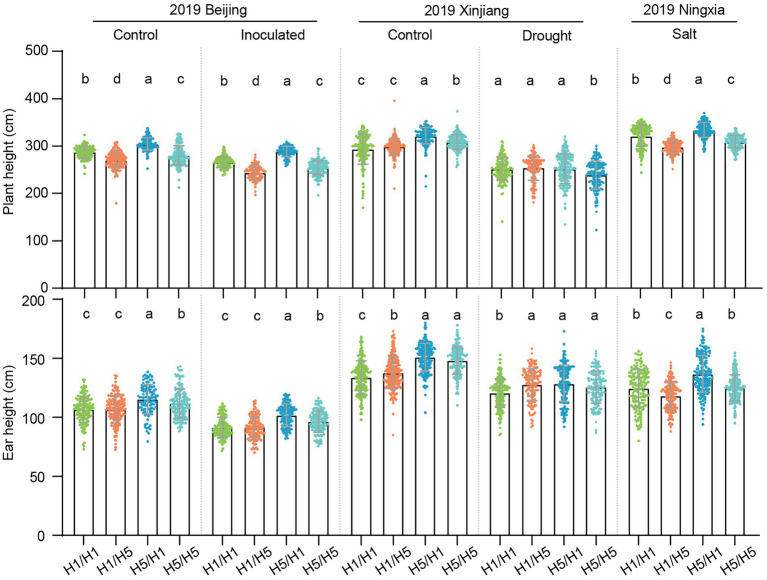
Plant architecture traits of hybrids in 2019 under control, inoculated, drought, and salt conditions. H1/H1, H1/H5, H5/H1, and H5/H5 are genotypes of four hybrids representing 83B28*^H1^* × A5302*^H1^*, 83B28*^H1^* × A5302*^H5^*, 83B28*^H5^* × A5302*^H1^*, and 83B28*^H5^* × A5302*^H5^*, respectively. Values are mean ± standard deviation (s.d.). Different letters indicate significant differences (*p* < 0.05, one-way ANOVA, Tamhane’s test, and Duncan’s test).

To better understand the genetic effects of haplotype *H5* on plant morphological traits, we investigated the plant height (PH) and ear height (EH) of the hybrids under different environmental conditions. In 2019, 83B28*^H5^* × A5302*^H1^* had significantly higher PH and EH than 83B28*^H1^* × A5302*^H1^* in Beijing (normal and infected conditions), Xinjiang (normal conditions), and Ningxia (salt stress; Figure 7). In Beijing and Xinjiang in 2020, 83B28*^H5^* × A5302*^H1^* and 83B28*^H5^* × A5302*^H5^* had significantly higher PH than 83B28*^H1^* × A5302*^H1^* and 83B28*^H1^* × A5302*^H5^* (Supplementary Figure 6). Finally, 83B28*^H5^* × A5302*^H1^* had significantly higher EH than 83B28*^H1^* × A5302*^H5^*, while the EH values of 83B28*^H5^* × A5302*^H5^* and 83B28*^H1^* × A5302*^H1^* fell between those of 83B28*^H5^* × A5302*^H1^* and 83B28*^H1^* × A5302*^H5^* (Supplementary Figure 6). In conclusion, haplotype *H5* significantly increased PH and EH in the hybrid 83B28*^H5^* × A5302*^H1^*.

## Discussion

### CCT domain genes are associated with crop yield

Increasing the resilience of crops is important in light of the changing climate (Juliana et al., 2019). Plant disease resistance usually compromises plant fitness (Brown, 2002; Yang et al., 2012; Li et al., 2016). The pleiotropic effects of *ZmCCT* on disease resistance and delayed flowering make it possible to have no yield penalty. We previously examined the performance of the progeny of a cross between 83B28*^H5/H1^* and A5302*^H4/H1^*, whose F_1_ hybrids could be divided into non-TE (*H4* and *H5*), TE (*H1*), and heterozygous genotypes at *ZmCCT* (Li et al., 2017b). This experiment did not clearly explain the genetic effects of *H5* or *H4*, and the test was only conducted for 1 year. In the current study, to explore the use of *H5* for maize resistance breeding, we obtained four hybrids by single crossing among two pairs of NILs: 83B28*^H1^*/83B28*^H5^* and A5302*^H1^*/A5302*^H5^*. The use of four hybrids allowed us to clearly observe the specific effects of different combinations of alleles under various conditions. The DSI values were significantly lower for 83B28*^H5^* × A5302*^H1^* and 83B28*^H5^* × A5302*^H5^* compared to the other two hybrids. Moreover, 83B28*^H5^* × A5302*^H1^* had significantly higher KWPE than the other three hybrids under both normal and infected conditions. In Xinjiang, 83B28*^H5^* × A5302*^H1^* and 83B28*^H5^* × A5302*^H5^* had higher KWPE values than the other two hybrids under well-watered conditions, while 83B28*^H5^* × A5302*^H1^* had no yield penalty compared to 83B28*^H1^* × A5302*^H1^* under drought stress. Overall, 83B28*^H5^* × A5302*^H1^* showed the best performance among the four hybrids.

*ZmCCT* expression is induced in roots in response to *F. graminearum*, confers stalk-rot resistance, and exhibits photoperiod sensitivity in leaves to delay flowering time (Yang et al., 2013; Wang et al., 2017). We propose that *ZmCCT* has different inducers in different tissues (leaves or roots), similar to the transcription factor gene *IPA1*. This may partly explain why the haplotype *H5* has no yield penalty compared to other disease resistance genes, or in some cases (like 83B28*^H5^* × A5302*^H1^*) even increases yield, like *IPA1* (Wang et al., 2018). The haplotype *H5* was slightly sensitive to photoperiod in the 83B28 and A5302 backgrounds; the underlying mechanism deserves further investigation. In wheat, the CCT domain gene *TaCol-B5* also has pleiotropic effects: It increases the number of spikelet nodes per spike, as well as the number of tillers and spikes, leading to increased grain yield (Zhang et al., 2022). CCT domain genes have obvious convergent effects in rice (*Ghd7*), maize (*ZmCCT*), and wheat (*TaCol-B5*), all of which are pleiotropic genes that regulate plant growth, development, stress responses, yield performance, and other agronomic traits (Xue et al., 2008; Weng et al., 2014; Zhang et al., 2022).

### 83B28*^H5^* × A5302*^H1^* and 83B28*^H1^* × A5302*^H5^* show opposite effects

Among the four hybrids, 83B28*^H5^* × A5302*^H1^* had better KWPE values than the others under both pathogen-infected and drought conditions, while 83B28*^H1^* × A5302*^H5^* showed better performance under salt stress conditions. In all circumstances, the effects of 83B28*^H5^* × A5302*^H1^* on yield-related traits were opposite those of 83B28*^H1^* × A5302*^H5^*. Whereas 83B28*^H5^* × A5302*^H1^* tended to show higher EL and KNPR values, 83B28*^H1^* × A5302*^H5^* tended to show higher KRN. In addition, the plant architecture traits of 83B28*^H5^* × A5302*^H1^* were opposite those of 83B28*^H1^* × A5302*^H5^*. Compared to 83B28*^H1^* × A5302*^H1^*, 83B28*^H5^* × A5302*^H1^* showed significantly greater PH, while 83B28*^H1^* × A5302*^H5^* generally showed lower PH. The flowering time of 83B28*^H5^* × A5302*^H1^* was significantly delayed in Beijing in 2020 and 2021, whereas that of 83B28*^H1^* × A5302*^H5^* was not. Why do these two hybrids exhibit such different characteristics even though they contain the same single haplotype *H5*? A recent study indicated that maternal and cytoplasmic effects influence multiple traits in durum wheat, such as resistance to *Zymoseptoria tritici*, plant height, and thousand kernel weight (Hassine et al., 2022). Therefore, perhaps, the haplotype *H5* has maternal effects on the hybrids.

### The relationships of flowering time, plant height, and yield

Alleles that delay flowering time generally increase plant height, such as *Ghd7* and *DTH8* (*Days to heading 8*) in rice (Xue et al., 2008; Wei et al., 2010), *gmap1* (homozygous quadruple mutant of *APETALA 1*) in soybean (Chen et al., 2020), and *lfy1* (*leafy* 1), *ZmCCT9*, and *ZmMADS69* (*MADS-box transcription factor*) in maize (Cui et al., 2017; Huang et al., 2018; Liang et al., 2019). In some cases, alleles that increase plant height also increase yield, as in *Ghd7* and *OsMPH1* (*MYB-like gene of plant height 1*; Zhang et al., 2017). It is reasonable to associate a longer vegetative stage with more biomass: The taller the plant, the higher the grain yield. However, in other cases, such as *zmm28* (AP1-FUL-Like MADS-Box Gene) in maize, increased yield is independent of plant height (Wu et al., 2019). In a study of 280 genotypic combinations within the same population, earlier flowering times were generally associated with reduced plant height and ear weight, although 11 optimal genotypic combinations were also identified (Xiao et al., 2021). In the current study, 83B28*^H5^* × A5302*^H1^* showed delayed flowering time under long-day conditions and increased PH and KWPE under all conditions except salt stress. These traits conferred by the haplotype *H5* must be intrinsically related.

### The haplotype *H5* of *ZmCCT* is a versatile maize resistance gene

In summary, the hybrid 83B28*^H5^* × A5302*^H1^* with a single haplotype *H5* showed an ~2-day delay in flowering time, increased stalk-rot resistance, and improved yield and yield-related traits under both biotic and abiotic stress conditions. From the perspective of maize breeding, an ~2-day delay in flowering time has little impact on farmers, but the yield and resilience of this crop under different conditions are important. Therefore, the haplotype *H5* is valuable due to its ability to optimize the trade-off between stress resistance and plant growth. However, the haplotype *H5* did not lead to better performance under salt stress, reminding us of the limits of using the haplotype *H5* in maize breeding.

In the current study, we also generated four hybrids from the cross between Zheng58*^H1^*/Zheng58*^H5^* and Chang7-2*^H1^*/Chang7-2*^H5^*. However, the hybrids with the haplotype *H5* exhibited much stronger photoperiod sensitivity than their parental lines and were therefore excluded from further study. While conducting this study, the haplotype *H5* was introgressed into a number of elite maize inbred lines in China. The stalk-rot resistance of these lines greatly improved, and grain yield routinely increased by 5%–10%. For example, the original maize variety MC812 (developed by the Beijing Academy of Agricultural and Forestry Sciences) had a diseased plant rate of 19.25% and a yield of 12,936 kg per hectare, in contrast to the improved MC812, with a diseased plant rate of 1.79% and a yield of 13,818 kg per hectare. In conclusion, the haplotype *H5* will be quite valuable for maize breeding for stalk-rot resistance and improved grain yield. This is a good example to exploit natural genetic variation to uncouple growth and defense trade-offs (He et al., 2022).

## Data availability statement

The original contributions presented in the study are included in the article/Supplementary material, further inquiries can be directed to the corresponding author.

## Author contributions

MX and LT designed the study, analyzed, and interpreted the data, and wrote and revised the manuscript. LT conducted the experiments. MY, MZ, and JY participated in field experiments and partial traits data collection. MZ helped in data analysis and data visualization. YL provided the seed resources. MX supervised the project. All authors contributed to the article and approved the submitted version.

## Funding

This study was supported by Jiangsu province’s Seed Industry Revitalization project [JBGS(2021)002] and Yunnan Provincial Science and Technology Department (202005AF150026).

## Conflict of interest

The authors declare that the research was conducted in the absence of any commercial or financial relationships that could be construed as a potential conflict of interest.

## Publisher’s note

All claims expressed in this article are solely those of the authors and do not necessarily represent those of their affiliated organizations, or those of the publisher, the editors and the reviewers. Any product that may be evaluated in this article, or claim that may be made by its manufacturer, is not guaranteed or endorsed by the publisher.
