# Supplementary material for: ZmCCT haplotype H5 improves yield, stalk-rot resistance, and drought tolerance in maize
Source: Front Plant Sci. 2022 Aug 15;13:984527. doi: 10.3389/fpls.2022.984527 (PMC9421135; doi:10.3389/fpls.2022.984527)
Supplement: Supplementary file 1 [file Data_Sheet_1.PDF]

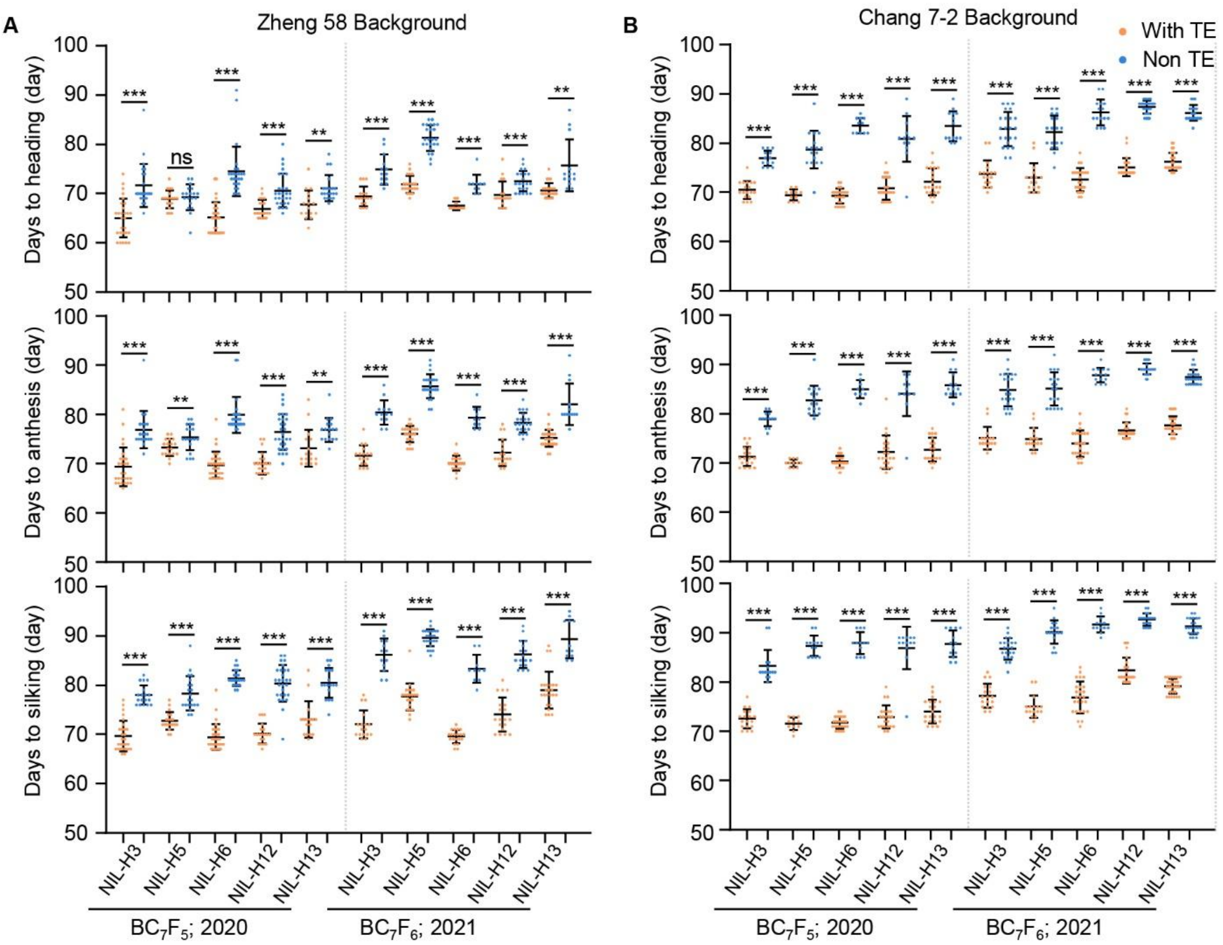

Supplementary Figure 1 | Twenty pairs of NILs in Zheng 58 **(A)** and Chang 7-2 **(B)** background were investigated for flowering-time in 2020 - 2021 Beijing under long-day conditions. The genotypes of every pairs of NILs were with TE *ZmCCT* homozygotes: *H1/H1*, and non-TE *ZmCCT* homozygotes: *H3/H3*, *H5/H5*, *H6/H6*, *H12/H12* or *H13/H13*. Values are mean ± standard deviation (s.d.). Asterisks indicate significant differences between *H1/H1* plants and the other genotypes (two-tailed paired Student's t-test, *, $P < 0.05$, **, $P < 0.01$, ***, $P < 0.001$, ns, not significant).

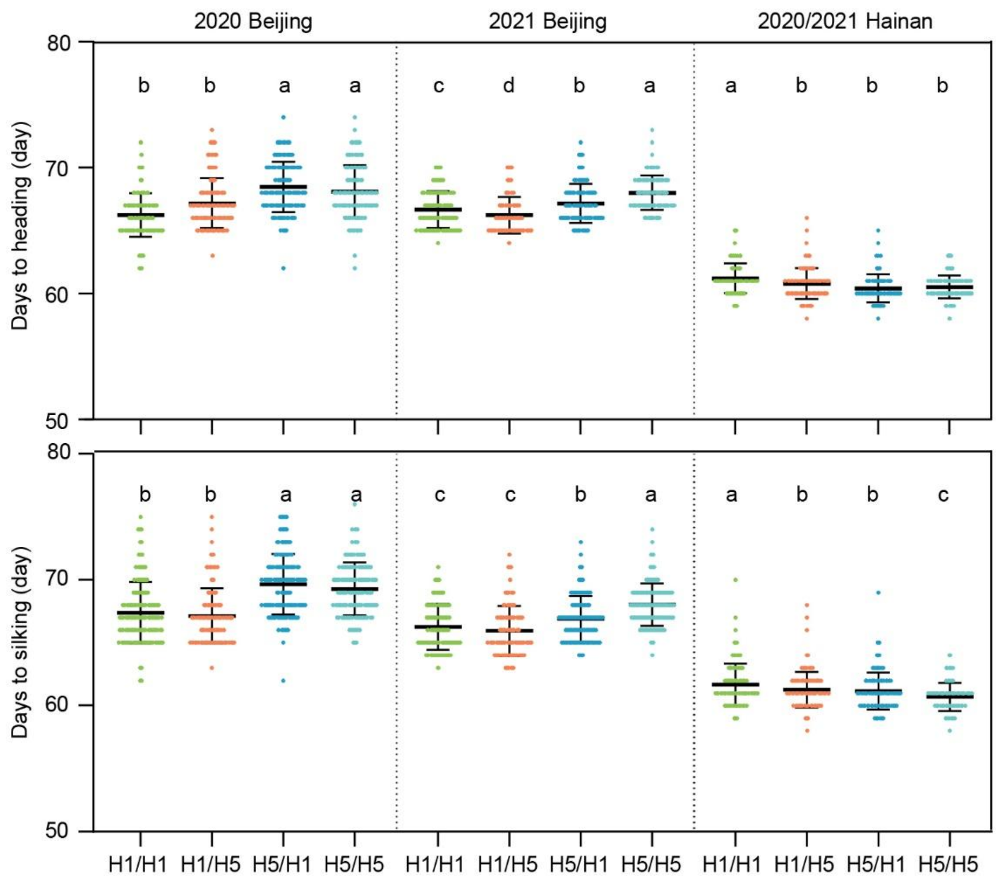

Supplementary Figure 2 | The flowering-time of four hybrids were investigated in 2020 - 2021 Beijing and 2020/2021 Hainan, which represents long-day conditions and short-day conditions, respectively. H1/H1, H1/H5, H5/H1 and H5/H5 are genotypes of four hybrids represent 83B28^{H1}×A5302^{H1}, 83B28^{H1}×A5302^{H5}, 83B28^{H5}×A5302^{H1} and 83B28^{H5}×A5302^{H5}, respectively. Values are mean ± standard deviation (s.d.). Different letters indicate significant differences among hybrids ($P < 0.05$, one-way ANOVA, Tamhane test).

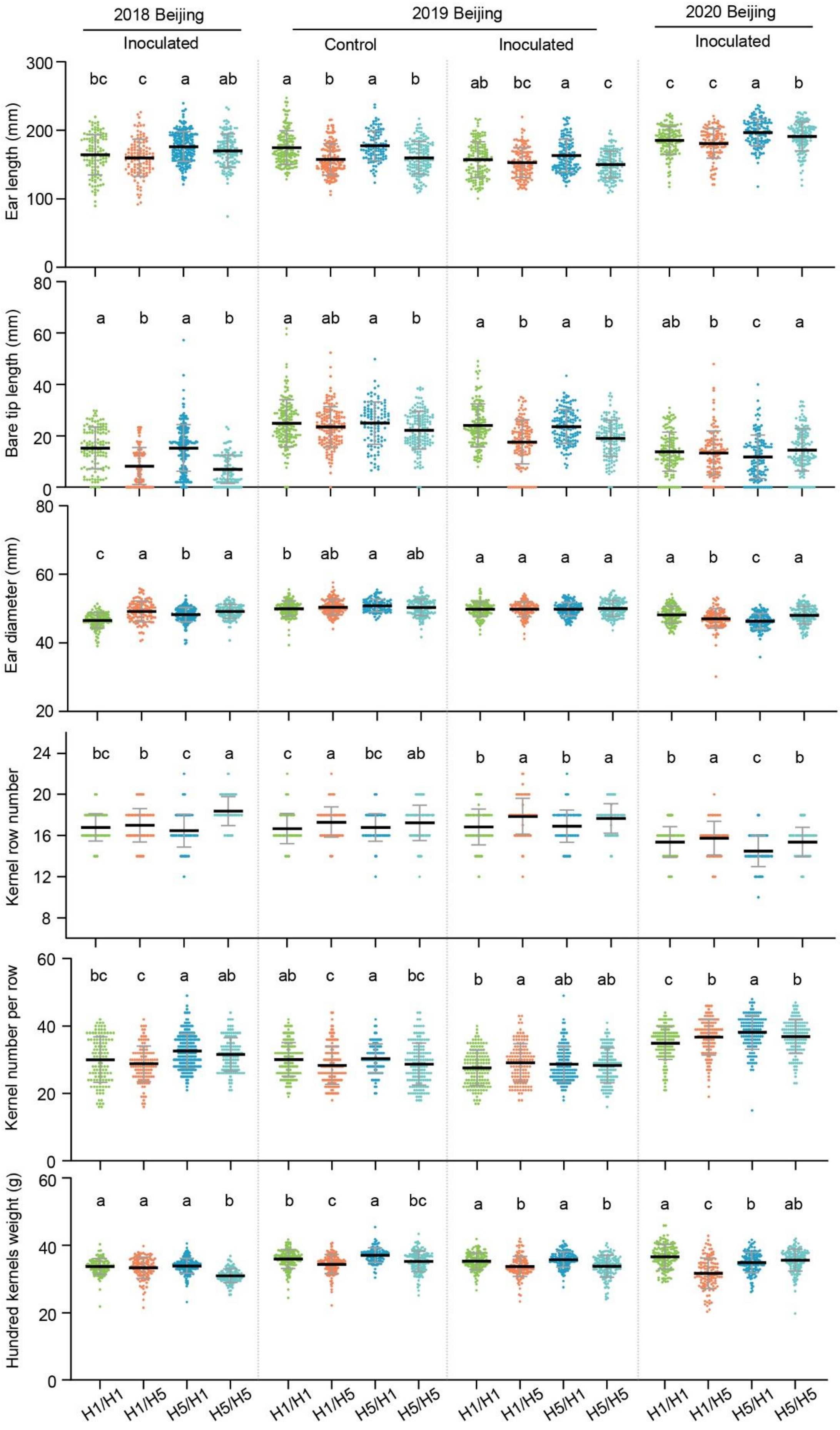

2018 Beijing
Inoculated
2019 Beijing
Control
Inoculated
2020 Beijing
Inoculated
Ear length (mm)
Bare tip length (mm)
Ear diameter (mm)
Kernel row number
Kernel number per row
Hundred kernels weight (g)
H1/H1
H1/H5
H5/H1
H5/H5

Supplementary Figure 3 | The yield related traits of hybrids in Beijing under control and biotic stress. Five traits contain ear length, ear diameter, kernel row number, kernel number per row and 100-kernels weight were evaluated in 2018 inoculated, 2019 control and inoculated, 2020 inoculated. H1/H1, H1/H5, H5/H1 and H5/H5 are genotypes of 4 hybrids represent 83B28^{H1}×A5302^{H1}, 83B28^{H1}×A5302^{H5}, 83B28^{H5}×A5302^{H1} and 83B28^{H5}×A5302^{H5}, respectively. Bars show mean, each spot represent an individual data. Different letters indicate significant differences among hybrids (P < 0.05, one-way ANOVA, Tamhane's test and Duncan's test).

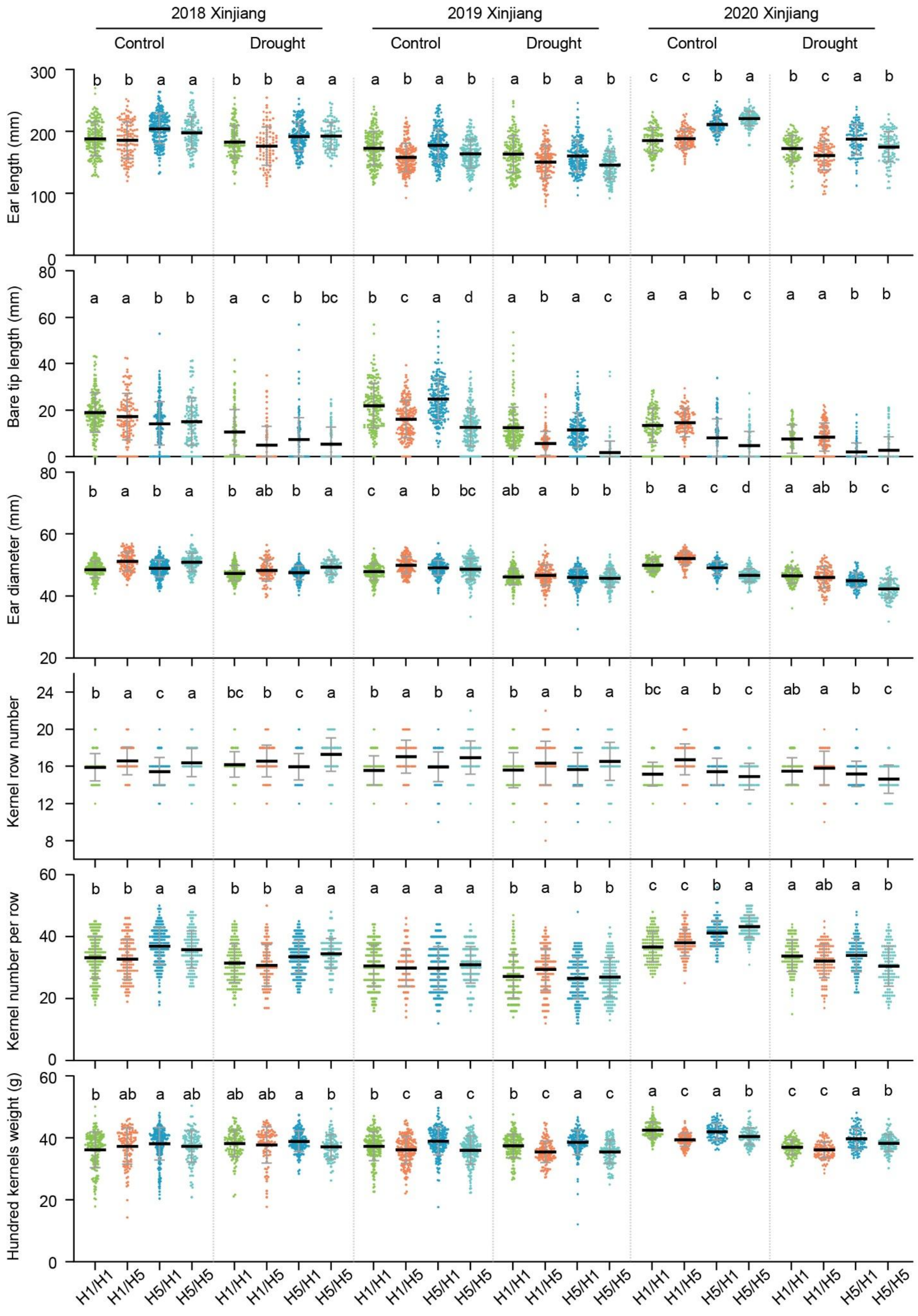

2018 Xinjiang
2019 Xinjiang
2020 Xinjiang
Control
Drought
Ear length (mm)
Bare tip length (mm)
Ear diameter (mm)
Kernel row number
Kernel number per row
Hundred kernels weight (g)
H1/H1
H1/H5
H5/H1
H5/H5

Supplementary Figure 4 | The yield related traits of hybrids in 2018 – 2020 Xinjiang under control and drought stress. Five traits contain ear length, ear diameter, kernel row number, kernel number per row and 100-kernels weight were evaluated. H1/H1, H1/H5, H5/H1 and H5/H5 are genotypes of 4 hybrids represent $83B28^{H1}$×$A5302^{H1}$, $83B28^{H1}$×$A5302^{H5}$, $83B28^{H5}$×$A5302^{H1}$ and $83B28^{H5}$×$A5302^{H5}$, respectively. Boxes show mean and quartile, whiskers represent max to min. Different letters indicate significant differences among hybrids ($P < 0.05$, one-way ANOVA, Duncan's test and Tamhane's test).

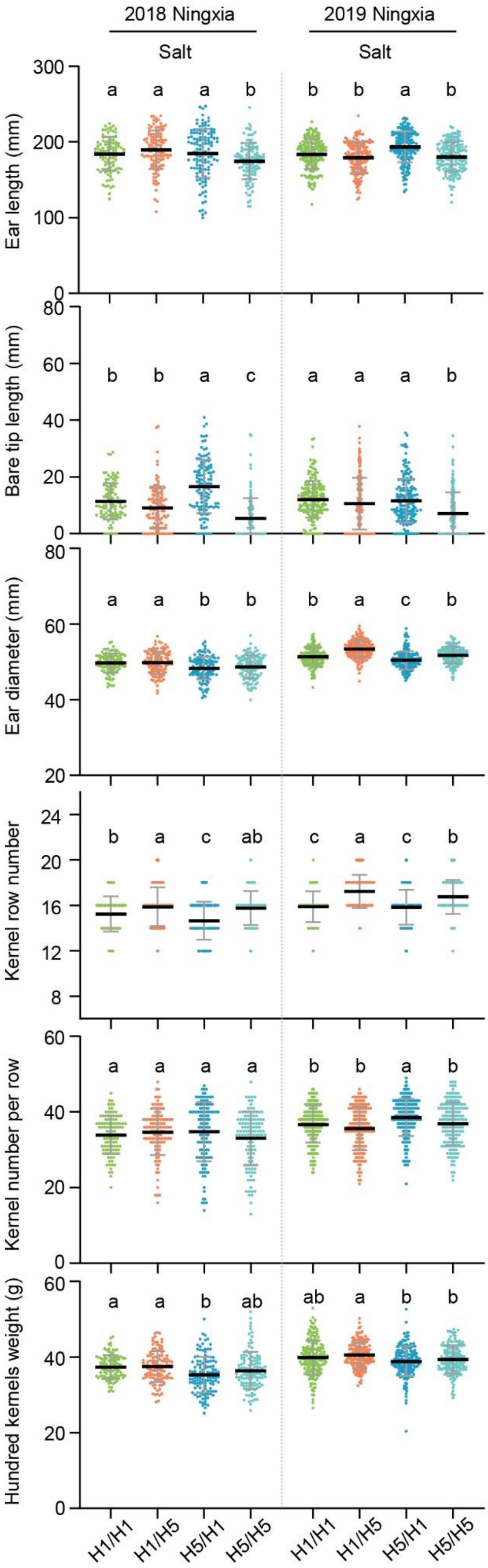

Supplementary Figure 5 | The yield related traits of hybrids in 2018 – 2019 Ningxia under salt stress

conditions. Five traits contain ear length, ear diameter, kernel row number, kernel number per row and 100-kernels weight were evaluated. H1/H1, H1/H5, H5/H1 and H5/H5 are genotypes of 4 hybrids represent 83B28^{H1}×A5302^{H1}, 83B28^{H1}×A5302^{H5}, 83B28^{H5}×A5302^{H1} and 83B28^{H5}×A5302^{H5}, respectively. Boxes show mean and quartile, whiskers represent max to min. Different letters indicate significant differences among hybrids (P < 0.05, one-way ANOVA, Duncan's test and Tamhane's test).

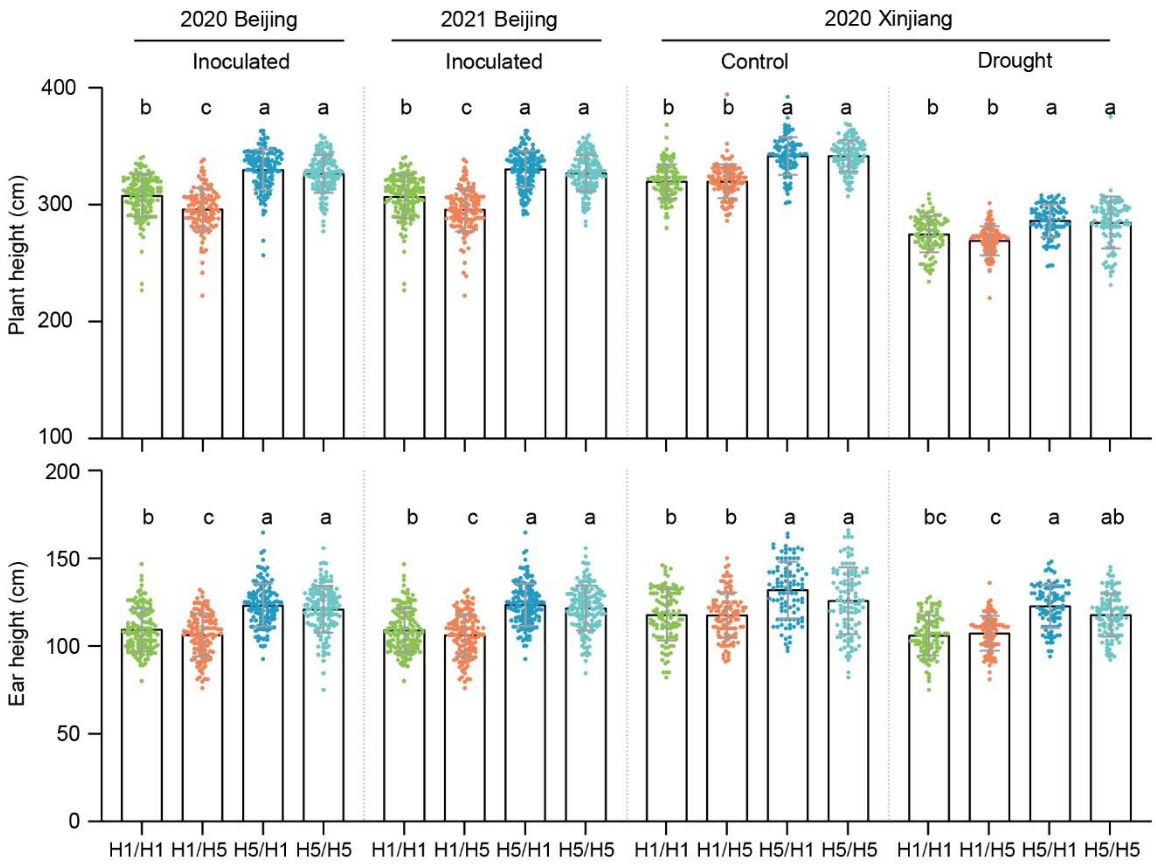

Supplementary Figure 6 | Plant architecture traits of hybrids under inoculated, control and drought stress conditions. H1/H1, H1/H5, H5/H1 and H5/H5 are genotypes of 4 hybrids represent 83B28^{H1}×A5302^{H1}, 83B28^{H1}×A5302^{H5}, 83B28^{H5}×A5302^{H1} and 83B28^{H5}×A5302^{H5}, respectively. Values are mean ± standard deviation (s.d.). Different letters indicate significant differences (P < 0.05, one-way ANOVA, Duncan's test and Tamhane's test).
